# Heat capacity peak at the quantum critical point of the transverse Ising magnet CoNb_2_O_6_

**DOI:** 10.1038/ncomms8611

**Published:** 2015-07-06

**Authors:** Tian Liang, S. M. Koohpayeh, J. W. Krizan, T. M. McQueen, R. J. Cava, N. P. Ong

**Affiliations:** 1 Department of Physics, Princeton University, Princeton, New Jersey 08544, USA; 2 Institute for Quantum Matter, Department of Physics and Astronomy, Johns Hopkins University, Baltimore, Maryland 21218, USA; 3 Department of Chemistry, Princeton University, Princeton, New Jersey 08544, USA; 4 Department of Chemistry, Johns Hopkins University, Baltimore, Maryland, 21218, USA

## Abstract

The transverse Ising magnet Hamiltonian describing the Ising chain in a transverse magnetic field is the archetypal example of a system that undergoes a transition at a quantum critical point (QCP). The columbite CoNb_2_O_6_ is the closest realization of the transverse Ising magnet found to date. At low temperatures, neutron diffraction has observed a set of discrete collective spin modes near the QCP. Here, we ask if there are low-lying spin excitations distinct from these relatively high-energy modes. Using the heat capacity, we show that a significant band of gapless spin excitations exists. At the QCP, their spin entropy rises to a prominent peak that accounts for 30% of the total spin degrees of freedom. In a narrow field interval below the QCP, the gapless excitations display a fermion-like, temperature-linear heat capacity below 1 K. These novel gapless modes are the main spin excitations participating in, and affected by, the quantum transition.

## Abstract (web summary)

The archetypal transverse Ising magnet CoNb_2_O_6_ possesses a set of collective spin modes near its quantum critical point. Here, the authors use heat capacity measurements to evidence the existence of Fermionic gapless spin excitations at the critical point.

## Graphical abstract

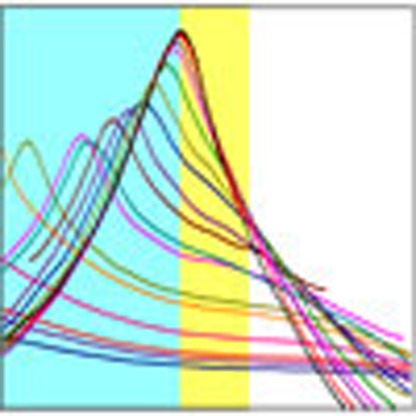

In the transverse Ising magnet (TIM), a magnetic field applied transverse to the easy axis of the spins induces a zero-Kelvin phase transition from the magnetically ordered state to the disordered state. Because it is the archetypal example of a system displaying quantum critical behaviour^1^, the TIM is prominently investigated in many areas of topical interest, for example, quantum magnetism^2^,^3^, integrable field theories^4^,^5^ and investigations of novel topological excitations^6^,^7^,^8^. The columbite CoNb_2_O_6_ is the closest realization found to date of the TIM in a real material. The spin excitations have been investigated by neutron diffraction spectroscopy near the quantum critical point (QCP)^3^ and in the paramagnetic state^9^, THz spectroscopy^10^ and ^93^Nb nuclear magnetic resonance^11^, but little is known about their thermodynamic properties at the QCP. Are there low-lying spin excitations distinct from the neutron-excited modes? What are their characteristics at the QCP?

Here we report a low-temperature heat capacity experiment that addresses these questions. We establish the existence of a large population of spin excitations that are gapless (after the phonon contribution is subtracted). As the transverse magnetic field is tuned towards the QCP, the spin heat capacity rises to a prominent peak. Below 1 K, the gapless modes display a temperature (*T*)-linear heat capacity similar to fermionic excitations. From the spin entropy, we infer that, at 1 K, the gapless modes account for 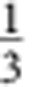 of the total spin degrees of freedom.

## Results

### Heat capacity versus temperature

**Figure 1:**
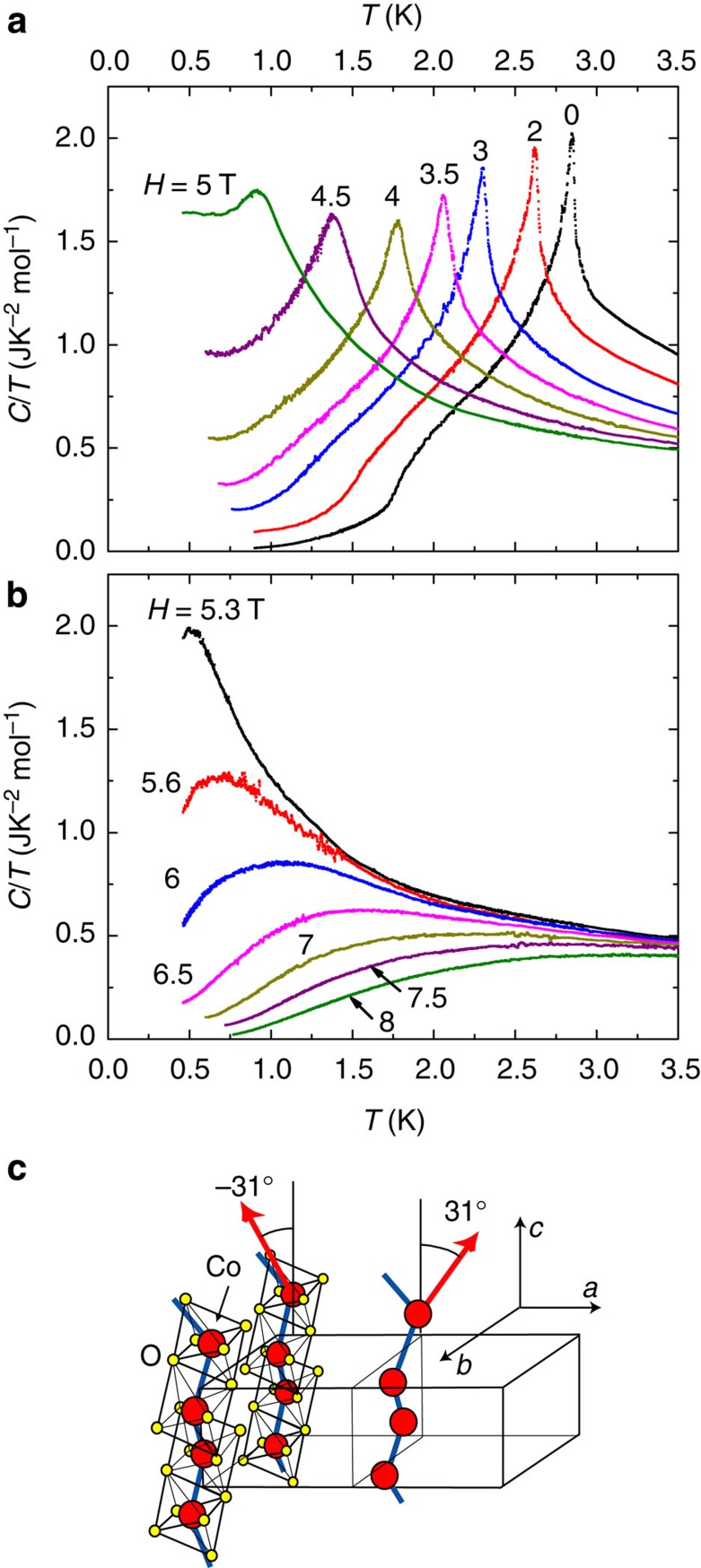
The heat capacity of CoNb_2_O_6_ versus temperature measured in a transverse magnetic field. The field **H** is applied parallel to the *b* axis. The quantity plotted is the heat capacity *C* divided by temperature *T*. (**a**) Curves of *C*/*T* versus *T* at fixed *H*<*H*_c_ (=5.24 T). In field *H*=0, a transition to an incommesurate phase occurs at *T*_c_(0)=2.85 K. With increasing *H*, the transition *T*_c_(*H*) is decreased. Below 1 K, *C*/*T* approaches saturation instead of decreasing to 0. At 5 T, *C*/*T* is *T* independent below 0.8 K. (**b**) Behaviour of *C*/*T* in the paramagnetic state (*H*>*H*_c_). Just above *H*_c_ (curve at *H*=5.3 T), *C*/*T* falls montonically as *T* increases above 0.5 K. For *H* slightly above 5.6 T, a field-dependent gap Δ appears. (**c**) the crystal structure of CoNb_2_O_6_. The easy axis (red arrows) is in the *a*–*c* plane at an angle ±31° to the *c* axis.

In CoNb_2_O_6_, the stacking of edge-sharing CoO_6_ octahedra along the *c* axis defines the Ising chain (inset in Fig. 1c). The isolated chain is described by the TIM Hamiltonian:


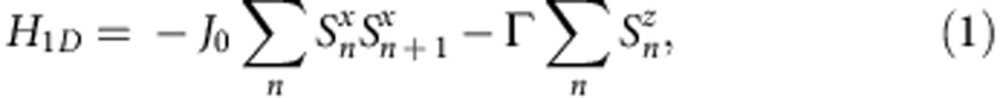


with *J*_0_ the ferromagnetic exchange along the easy axis **c**||**x** and Γ the transverse field. *S*_*n*_^*x*^ and *S*_*n*_^*z*^ are, respectively, the *x* and *z* components of the spin operator at lattice site *n*. In the *a*–*b* plane, the chains assume a triangular coordination^12^,^13^,^14^, with antiferromagnetic interactions |*J*_1_|, |*J*_2_|<<*J*_0_ between adjacent chains. Geometric frustration effects lead to competing antiferromagetic and ferrimagnetic ground states^14^. In a magnetic field **H**||**b**, CoNb_2_O_6_ exhibits a sharp transition to a three-dimensional (3D)-ordered phase at a critical temperature *T*_c_(*H*) that decreases from 2.85 K (at *H*=0) to zero as *H*→*H*_c_.

**Figure 2:**
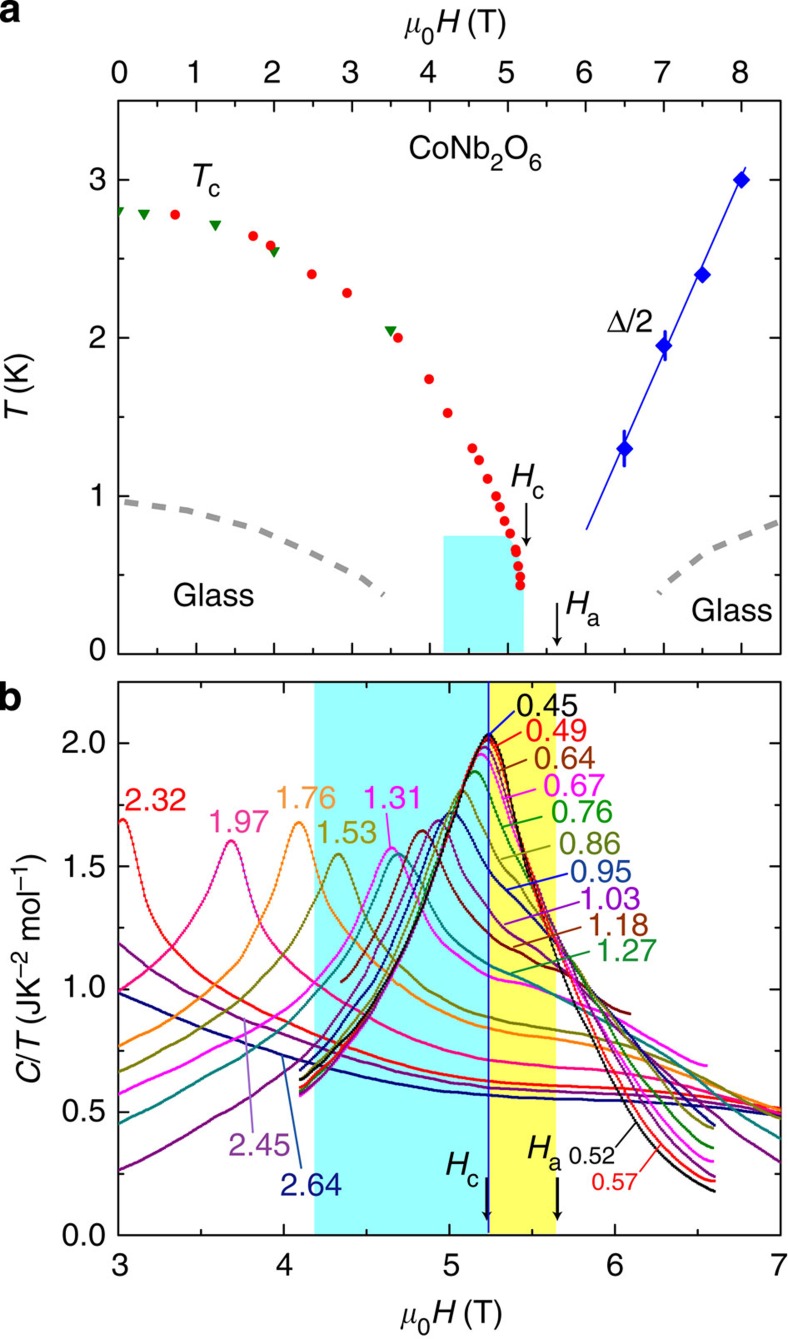
Phase diagram of CoNb_2_O_6_ in a transverse magnetic field and heat capacity peak at the QCP. (**a**) The phase diagram inferred from *C*/*T*. The transition *T*_c_(*H*) defines the ordered phase (solid circles and triangles represent *T*-constant and *H*-constant measurements, respectively). Gapless excitations with *T*-independent *C*/*T* are observed in the blue shaded region (4.2 T<*H*<*H*_c_). The gap Δ above *H*_a_ (solid diamonds) is inferred from fits to the one-dimensional exact solution. The error bars are estimated from the goodness of the fits at each *H*. *H*_*a*_ is the crossover field at which d(*C*/*T*)/d*T* changes sign at low *T*. The dashed curves are nominal boundaries of the low-*T* phases in which glassy behaviour is observed. (**b**) Curves of *C*/*T* measured versus *H* at constant *T*. Above ∼1 K, *C*/*T* climbs to a sharp peak when *H* crosses the boundary *T*_c_(*H*), and then falls monotonically. Below 1 K, however, the constant-*T* contours lock to the left branch of the critical peak profile (*C*/*T*)_0_ measured at 0.45 K (which peaks at *H*_c_=5.24 T). The locking implies *C*/*T* is *T*-independent below 0.8 K. In both panels, the blue shaded regions represent the field interval within which the locking is observed. Above *H*_c_, the contours are well separated at all *T*. The derivative d(*C*/*T*)/d*T* at low *T* is negative for *H*_c_<*H*<*H*_a_ (yellow region), but positive for *H*>*H*_a_.

To investigate the low-energy spin excitations in CoNb_2_O_6_, we have measured its low-temperature heat capacity *C*(*T*,*H*) by a.c. calorimetry over the *T*–*H* plane (see Methods). First, we discuss the curves of the heat capacity *C* versus *T* measured in fixed *H*. Figure 1a plots these curves as *C*/*T*(*T*,*H*) versus *T* for *H*<*H*_c_=5.24 T. In each curve, *C*/*T* displays a prominent peak when *T* crosses *T*_c_(*H*). In zero *H*, *C*/*T* decreases steeply below *T*_c_(0), and approaches zero at 1 K, consistent with the existence of a full gap. The shoulder feature near 1.7 K signals the transition from an incommensurate to commensurate AF (antiferromagnetic) phase^13^. At finite *H*, we observe significant enhancement of *C*/*T* throughout the ordered phase. Instead of falling to zero, the curves become *T* independent at low *T* (curve at 5 T). Between 4 and 5 T, the saturation value increases by more than a factor of 3. In the disordered phase (*H*>*H*_c_), we observe a profile that also reveals a gap Δ, but one that increases sharply with the reduced field *H*−*H*_c_ (Fig. 2a).

### Heat capacity versus field

To supplement the constant-*H* curves in Fig. 1, we performed measurements of *C*/*T* versus *H* at constant *T*. Figure 2a shows the phase diagram obtained from combining the constant-*H* and constant-*T* curves. The boundary of the ordered phase, *T*_c_(*H*) defined by the sharp peak in *C*/*T*, falls to zero as *H*→*H*_c_ (solid circles and triangles). Above *H*_c_, the gap Δ in the disordered phase (solid diamonds) is estimated from fits to the free-fermion solution discussed below. The dashed curves are the nominal boundaries below which glassy behaviour is observed (see below).

Figure 2b displays the constant-*T* scans in the region close to the QCP. If the temperature is fixed at a relatively high value, for example, *T*=1.76 K, *C*/*T*(*T*,*H*) initially rises to a sharp peak as *H* is increased from 0 to 4.1 T. Above 4.1 T, *C*/*T* falls monotonically with no discernible feature at *H*_c_. As we lower *T*, the peak field shifts towards *H*_c_=5.24 T, tracking *T*_c_(*H*) in the phase diagram in Fig. 2a. Below 1.5 K, the constant-*T* contours converge towards the prominent profile measured at 0.45 K, which is our closest approximation to the critical peak profile (*C*/*T*)_0_ at *T*=0. The contours under this profile reveal a remarkable structure. On the low-field side of the peak (4.2 T<*H*<*H*_c_), shaded blue in Fig. 2a,b, the contours lock to the critical peak profile as *T* decreases. This implies that, if *H* is fixed inside this interval, *C*/*T* assumes the *T*-independent value (*C*/*T*)_0_ at low *T*. Hence the *T*-independent plateau seen in the curve at 5 T in Fig. 1a is now seen to extend over the entire blue region. When *H* exceeds *H*_c_, however, the locking pattern vanishes. The different *T* dependencies reflect the distinct nature of the excitations on either side of *H*_c_. (Slightly above *H*_c_, the derivative d(*C*/*T*)/d*T* changes from negative to positive at a crossover field *H*_a_∼5.6 T.)

### Spectrum of *C*_eff_ and glassy response

In a.c. calorimetry, the spectrum of the effective (observed) heat capacity *C*_eff_(*ω*) varies in a characteristic way with the measurement frequency *ω*. For each representative local region of the *T*–*H* phase diagram investigated, we measured 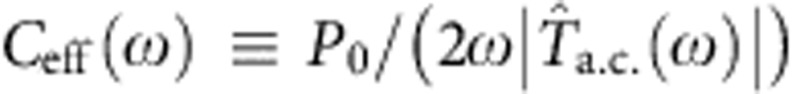 over the frequency range 0.02–100 Hz, where *P*_0_ is the applied power and 
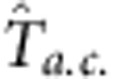
 is the complex temperature (see Methods). The spectrum of *C*_eff_(*ω*) has a hull-shaped profile characterized by the two characteristic times *τ*_1_ (set by the sample's parameters) and *τ*_ext_ set by coupling with the bath (defined in Methods). In both the low-*ω* and high-*ω* regions (*ω*<<1/*τ*_ext_ and *ω*>>1/*τ*_1_, respectively) *C*_eff_(*ω*) rises steeply above the true (equilibrium) heat capacity *C*. However, there exists a broad frequency range in between where *C*_eff_(*ω*) is nearly *ω*-independent and equal to the intrinsic equilibrium heat capacity *C* of the sample. All results reported here are taken with *ω* within this sweet spot. Within the regions denoted as glassy in Fig. 2, the spectrum is anomalous (Methods). Instead of the hull-shaped spectrum, the measured *C*_eff_ decreases monotonically over the accessible frequency range. We define these regions of the phase diagram as glassy. We note that the QCP region lies well away from the glassy regions.

### Temperature-linear heat capacity at critical field

**Figure 3:**
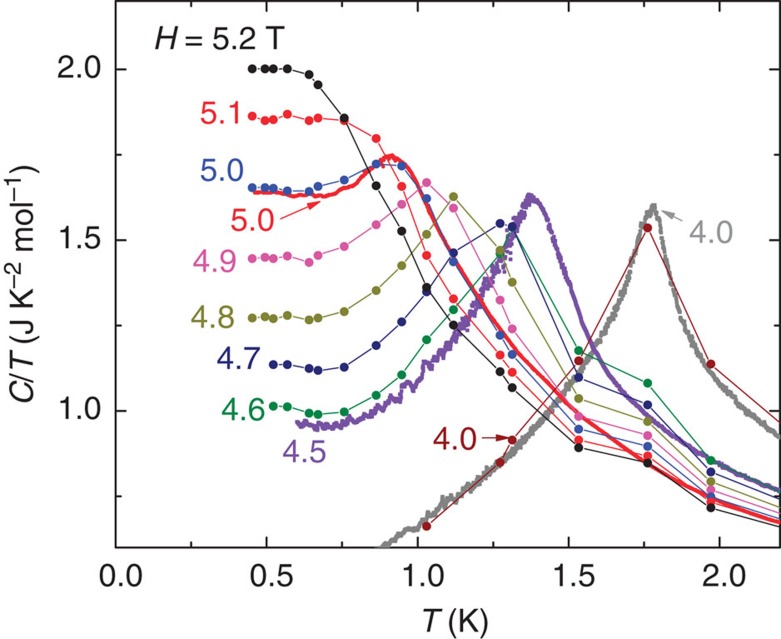
Low-temperature heat capacity and spin entropy in expanded scale near the critical field. The discrete symbols (solid circles) are values of *C*/*T* extracted from continuous measurements of *C*/*T* versus *H* at fixed *T* (the constant-*T* scans plotted in the phase diagram). Here they are plotted versus *T* at fixed *H* to bring out the fixed-field contours. Below 0.8 K, the values of *C*/*T* saturate to a *T*-independent value that depends on *H*. These plateau values occur within the region of the phase diagram where the contours display locking behaviour. To supplement the discrete data points, we also measured *C*/*T* continuously versus *T* at fixed *H*. These curves are shown as continuous curves at *H*=4.0, 4.5 and 5.0 T. The agreement between the two distinct experiments is very close.

To make explicit the *T*-independent behaviour below *H*_c_, we have extracted the values of *C*/*T*(*T*,*H*) and replotted them in Fig. 3 as constant-*H* curves for eight values of *H* between 4 and 5.2 T. As is evident, the curves approach a constant value when *T* decreases below 0.8 K. The flat profiles reflect the locking of the contours described above. We have also plotted the constant-*H* curves measured at 4, 4.5 and 5 T (continuous curves) to show the close agreement between the two sets of data. The critical peak profile in Fig. 2b and the *T*-independent contours shown in Fig. 3 are our key findings in this report.

The *T*-linear behaviour of *C* at low *T* illuminates the nature of the low-lying excitations. For fermions, *C* is linear in *T* and given by 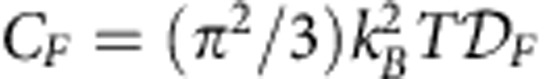, where 
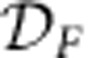
 is the density of states at the Fermi level. The Hamiltonian equation (1) can be diagonalized by transforming to free fermions^15^,^16^ (see Free-fermion solution in Methods). The heat capacity of the isolated Ising chain may then be calculated^17^. The issue whether the free fermions are artifacts or real observables is currently debated^6^,^7^. However, our system is 3D with finite *J*_1_ and *J*_2_. To our knowledge, fermionic excitations in the ordered phase have not been anticipated theoretically.

### Low-temperature spin entropy at critical field

We next show that the critical peak profile ((*C*/*T*)_0_ in Fig. 2b) accounts for a surprisingly large fraction of the total spin degrees of freedom (d.o.f.). Before extracting the spin entropy from the measured *C*, we need to subtract the phonon contribution. Fortunately, the spin contributions may be readily distinguished from the phonon term. Following the procedure of Hanawa *et al*.^12^, we have carried out this subtraction to isolate the spin part of the heat capacity *C*_s_(*T*,*H*) (see Methods). The spin entropy is then given by the integral 
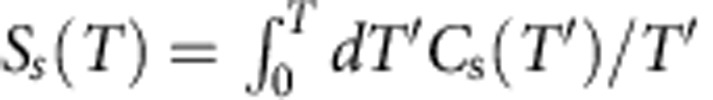
.

**Figure 4:**
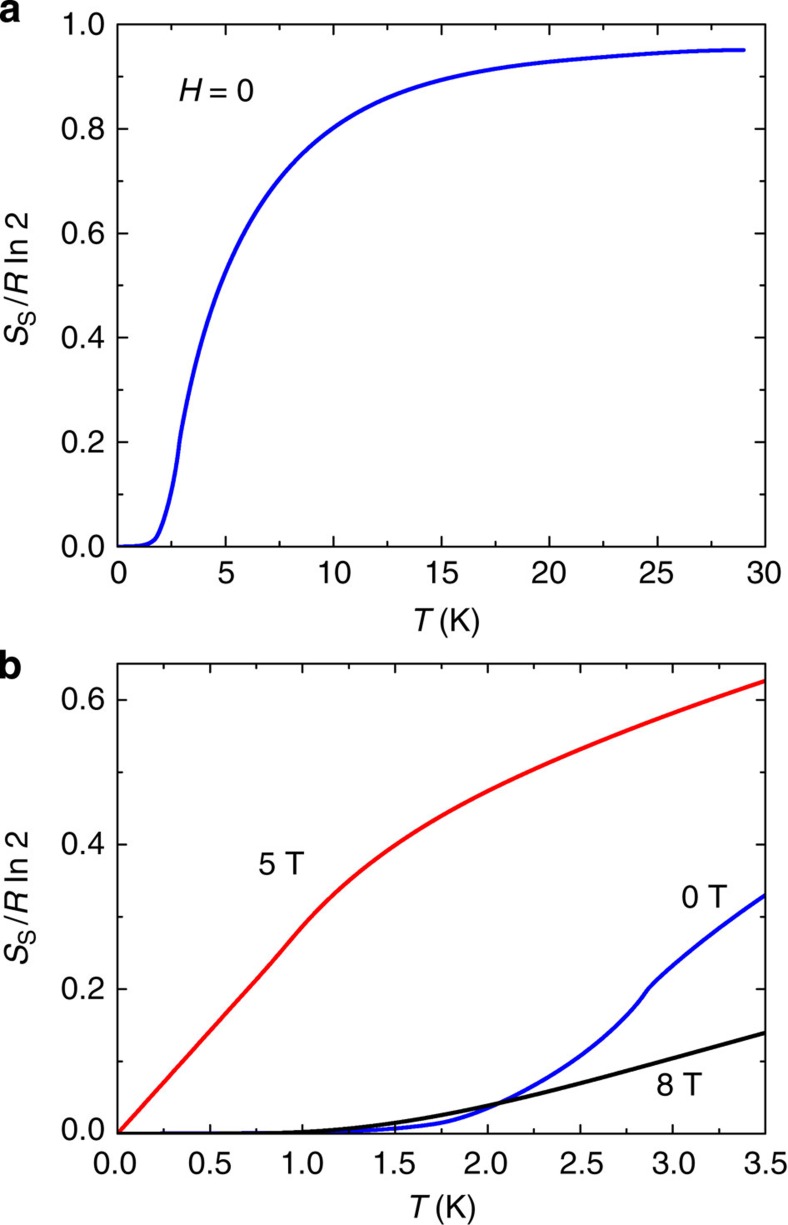
The spin entropy of CoNb_2_O_6_ derived from its heat capacity. To obtain the spin entropy *S*_s_, we first subtract the phonon contribution from the measured heat capacity to isolate the spin contribution *C*_s_. The spin entropy *S*_s_(*T*) is then obtained by integrating *C*_s_(*T*)/*T* with respect to *T*. (**a**) The profile of *S*_s_ in zero magnetic field. At 20 K, 90% of the spin entropy frozen out at low *T* is recovered. Although our measurements extend only to 30 K, the curve of *S*_s_ is expected to asymptote to 1 above room temperature. (**b**) The spin entropy *S*_s_ versus *T* at *H*=0, 5 and 8 T. *S*_s_ is obtained by integrating the curves of *C*_s_/*T* after subtracting the phonon contribution (derived from the results of Hanawa *et al*. on the nonmagnetic analogue ZnNb_2_O_6_). At 5 T, *S*_s_ accounts for nearly 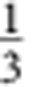 of the spin d.o.f. at 1 K.

First, we verified that, at *H*=0, the curve of *S*_s_(*T*) obtained by integrating *C*_s_/*T* rises rapidly above 20 K to closely approach the value *R* ln 2 (*R* is the universal gas constant), thus accounting for the total spin d.o.f. By integrating *C*_s_/*T* with respect to *T*, we obtain the total spin entropy *S*_s_(*T*). In Fig. 4a, the variation of *S*_s_(*T*) versus *T* inferred from the data at zero *H* is plotted. Above ∼5 K, *S*_s_ rises rapidly attaining 90% of *R* ln 2 by 20 K.

Our interest here is the behaviour of *S*_s_ at low *T*, which we plot in Fig. 4b. In contrast to *S*_s_(*T*) at *H*=0 and 8 T, the curve for *S*_s_(*T*) at 5 T is strongly enhanced and varies linearly with *T* with a slope equal to *C*_s_/*T*. As *H*→*H*_c_, the spin entropy rises to ∼30% of *R* ln 2 at 1 K. Hence, the gapless excitations account for nearly 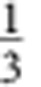 of the total spin d.o.f.

### Paramagnetic state heat capacity and the free-fermion solution

In the paramagnetic state above *H*_c_, it is instructive to compare the measured *C*_s_/*T* with the heat capacity calculated from the free energy in the free-fermion solution 17 (see Methods) given by


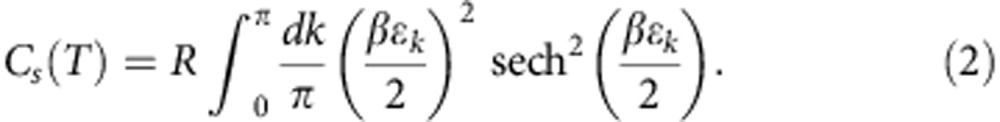


where *R* is the gas constant, *β*=1/(*k*_B_*T*), and the energy of the fermions is 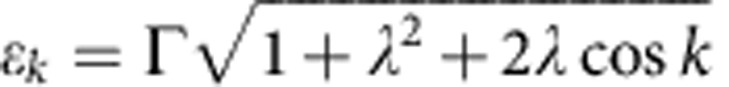, with *λ*=*J*_0_/2Γ. For each field *H*>*H*_c_, we took Γ and *λ* as adjustable parameters (*J*_0_ is fixed at 21.4 K for all *H*).

**Figure 5:**
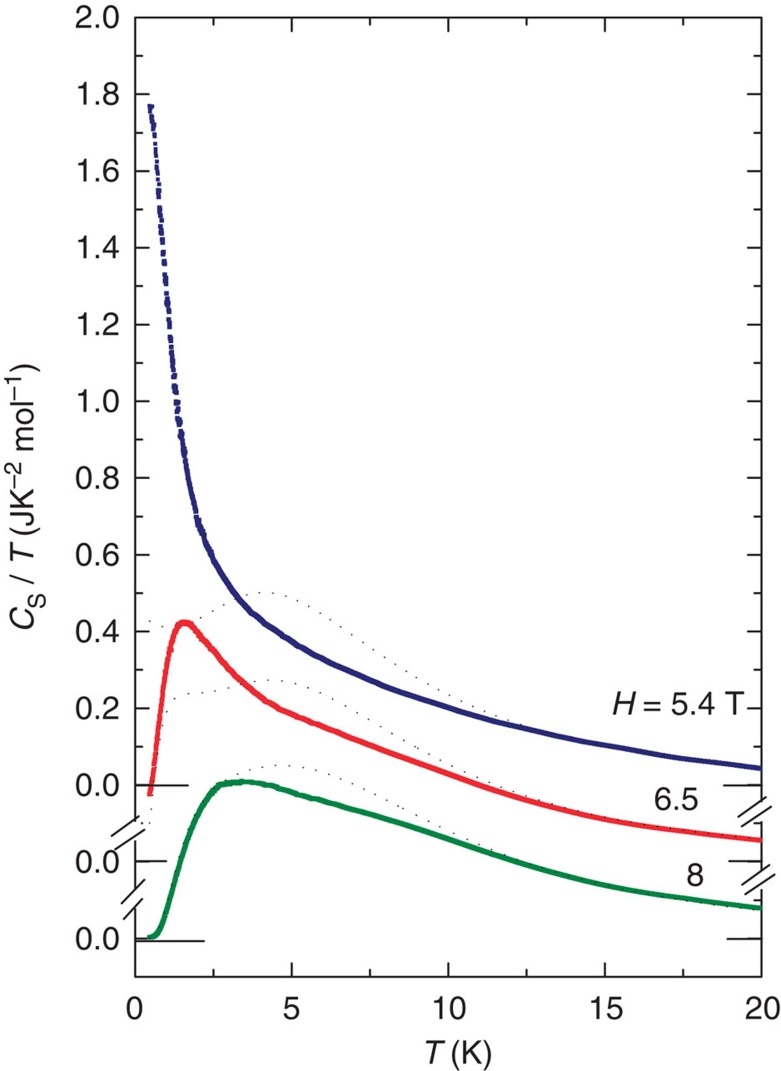
The heat capacity in the paramagnetic phase at three values of magnetic field above the critical value. The phonon contribution to the measured *C* has been subtracted. For clarity, the curves have been displaced vertically. The dashed curves are fits to the free-fermion solution using the values Γ=11, 13.24 and 16.3 K for the curves at *H*=5.4, 6.5 and 8 T, respectively. The corresponding values of *λ* are 0.97, 0.806 and 0.655. The value of *J*_0_ is fixed at 21.4 K. Deviations from the fits become pronounced as *H*→*H*_c_.

As shown in Fig. 5, the fits (dashed curve) are reasonable only above 10 K. Below 10 K, deviations become increasingly prominent as we lower *H* towards *H*_c_. In particular, the striking divergence of the curve at *H*=5.4 T (as *T*→0) lies well beyond the reach of equation (2). These deviations reveal that the incipient magnetic ordering effects extend deep into the paramagnetic phase. The curves in Figs 2 and 3 reveal how these deviations smoothly evolve into the gapless excitations. Models that include interchain exchange terms (for example, as proposed in ref. 9) are more realistic. Comparison of our data with the behaviour of the *C*_s_ predicted near the QCP should be highly instructive. To our knowledge, solutions in the quantum regime have not been reported.

### Negligible contribution from nuclear spin degrees

We discuss whether contributions of the nuclear spins to the heat capacity play any role in the experiment. The nuclear spins contribute as a Schottky term given by^18^ *C*_*N*_=*Nk*_B_*X*^2^e^*X*^/(e^*X*^+1)^2^ (for the two-level case), where *X*=Δ*E*/*k*_B_*T*, and Δ*E* is the energy splitting of the levels. *C*_*N*_ peaks near Δ*E*/*k*_B_ (typically 10–30 mK^18^,^19^) and falls off as (Δ*E*/*k*_B_*T*)^2^≡*A*/*T*^2^ for *T*>>Δ*E*/*k*_B_. The most favourable situation is when *H* increases Δ*E* by the Zeeman energy, which is Δ*E*=*μμ*_*N*_*H*, where *μ* is the nuclear moment and *μ*_*N*_=0.37 mK T^−1^ the nuclear Bohr magneton. We have *μ*=6.17 for ^93^Nb and 4.63 for ^59^Co. The larger moment gives *A*=0.032 mJK mol^−1^ at 5 T. At *T*=1 K, this yields values for *C*_*N*_ that are extremely small (by a factor of 10^5^) compared with *C* displayed in Figs 1 and 3. Hence, the nuclear spin d.o.f. cannot be resolved in our experiment.

## Discussion

In the isolated Ising chain, the excitations are domain walls (kinks) that separate degenerate spin-↑ from spin-↓ domains. In our 3D system, the self-consistent fields derived from *J*_1_ and *J*_2_ lift the degeneracy. As a result, kinks and antikinks interact via a linear potential (the energy cost of the unfavoured domain) to form bound pairs^20^,^21^. The quantized excitations of the bound pairs have been detected by neutron diffraction spectroscopy^3^ and by time-domain THz spectroscopy^10^ as discrete modes (the lowest mode has energy 1.2 meV at *H*=0 and 0.4 meV at 5 T). As *H*→*H*_c_, the ratio of the two lowest modes approaches the golden ratio, consistent with the E8-Lie group spectrum^3^. However, these modes are too high in energy to contribute to *C*/*T* below 1 K. Rather, our experiment provides firm evidence for a band of low-lying, gapless spin excitations that are entirely distinct from the high-energy modes. The steep increase of the spin entropy *S*_s_ at 5 T (Figure 4b) shows that *S*_s_ has attained 30% of its high-*T* value already at 1 K. Thus, the large anomalous peak centred at the QCP accounts for a significant fraction 
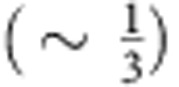
 of the total spin d.o.f. In addition to the remarkable spin modes observed at discrete energies by neutron diffraction spectroscopy and THz spectroscopy, a substantial fraction of the spin d.o.f. exists as (essentially) gapless modes, which peak in weight at the QCP. How the two sets of excitations co-exist is a problem that confronts the theoretical description of the QCP in this material.

Perhaps, the most surprising finding from the experiment is the *T*-independent profile of *C*_s_(*T*)/*T* below 1 K in the field interval 4.2<*H*<*H*_c_ abutting *H*_c_. The results imply that the excitations obey Fermi–Dirac statistics. In the one-dimensional TIM model, the solution obtained via the Jordan–Wigner transformation (see Methods) yield free fermions. However, as mentioned above, it is uncertain whether these fermions are physically observable. Moreover, the interchain exchange in the real material^9^ may render the free-fermion solutions inapplicable. The present finding that the *T*-linear behaviour is confined to the QCP region where *C*/*T* displays a prominent peak highlights serious gaps in our understanding of the QCP and the effects of strong quantum fluctuations in its vicinity. The heat capacity invites a detailed investigation of the quantum behaviour at the QCP in realistic models applicable to CoNb_2_O_6_.

The heat capacity experiment shows that, in the vicinity of the QCP, the gapless modes constitute the dominant spin excitations that are affected by the quantum transition induced by the applied transverse *H*. As seen in the set of curves in Fig. 2b, the QCP strongly affects *C*/*T* versus *H* to produce a profile that, at 0.45 K, rises to a prominent peak at the critical field. We reason that the gapless modes are the relevant modes that participate in the quantum transition at *H*_c_. The dominant fluctuations associated with the quantum transition are inherent to these modes. From the spin entropy, we infer that they account for nearly 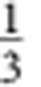 of the total spin degrees of freedom in the sample. As discussed, the gapless modes display a fermion-like heat capacity below 1 K over a broad region of the ordered phase below *H*_c_.

## Methods

### Crystal growth

The CoNb_2_O_6_ powder was packed and sealed into a rubber tube evacuated using a vacuum pump. The powder was then compacted into a rod, typically 6 mm in diameter and 70-mm long, using a hydraulic press under an isostatic pressure of 7 × 10^7^ Pa. After removal from the rubber tube, the rods were sintered in a box furnace at 1,375° C for 8 h in air.

Single crystals of ∼5 mm in diameter and 30 mm in length were grown from the feed rods in a four-mirror optical floating zone furnace (Crystal System Inc. FZ-T-4000-H-VII-VPO-PC) equipped with four 1-kW halogen lamps as the heating source. In all the growth processes, the molten zone was moved upwards with the seed crystal being at the bottom and the feed rod above it. Growths were carried out under 2 bar O_2_–Ar (50/50) atmosphere with the flow rate of 50 ml min^−1^, at the zoning rate of 2.5 mm h^−1^, with rotation rates of 20 r.p.m. for the growing crystal (lower shaft) and 10 r.p.m. for the feed rod (upper shaft). In all runs, only one zone pass was performed.

Phase identification and structural characterization were obtained using a Bruker D8 Focus X-ray diffractometer operating with Cu K radiation and Lynxeye silicon strip detector on finely ground powder from the crystal boules, while back-reflection X-ray Laue diffraction was utilized to check the crystalline qualities and orientations of the crystals. Measurements were carried out on oriented thin rectangular-shaped samples cut directly from the crystals using a diamond wheel.

### A.C. calorimetry

The heat capacity was measured using the a.c. calorimetry technique^22^ on a crystal of CoNb_2_O_6_ (∼1 × 3 × 0.5 mm along the *a*, *b* and *c* axes, respectively) in a magnetic field *H* applied along the *b* axis. Using an a.c. current of frequency 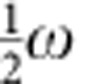, we applied the a.c. power (*P*_0_/2)exp(*iωt*) to the sample via a 1-kΩ RuO_2_ thin-film resistor (*P*_0_ ranged from ∼0.4 to 5 μW for measurements below 4 K, and ∼5 to 400 μW from 4 to 30 K). The (complex) temperature 
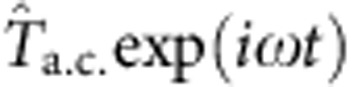
 was detected at the thermometer (a 20-kΩ RuO_2_ thin-film resistor). In the slab geometry of ref. 22, 
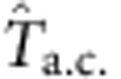
 is given by


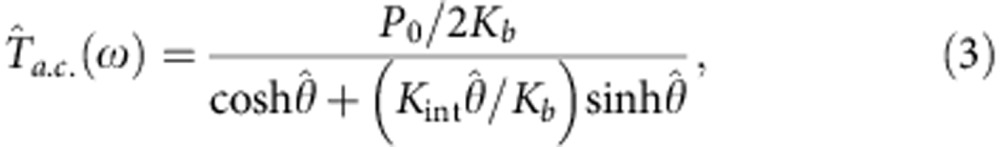


where 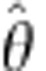 is the complex angle


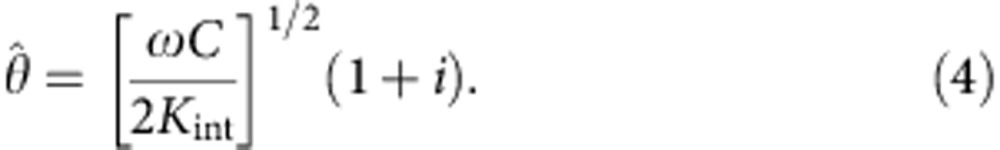


Here, *K*_int_ is the thermal conductance of the sample, *K*_b_ the thermal conductance between the sample and the thermal bath and *C* the heat capacity of the sample.

Taking into account that the thermal conductance between the thermometer and the sample, heater and the sample is finite, and under the condition that the internal relaxation time constant 
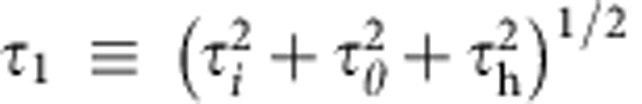
 (where *τ*_*i*_=*C*/6*K*_int_, *τ*_*θ*_=*C*_*θ*_/*K*_*θ*_, *τ*_h_=*C*_h_/*K*_h_, *C*_*θ*_ heat capacity of the thermometer, *C*_h_ heat capacity of the heater, *K*_*θ*_ thermal conductance between the thermometer and the sample, *K*_h_ thermal conductance between the heater and the sample) and the external relaxation time constant *τ*_ext_≡*C*/*K*_b_ satisfy *ωτ*_1_<<1<<*ωτ*_ext_, equation (3) reduces to


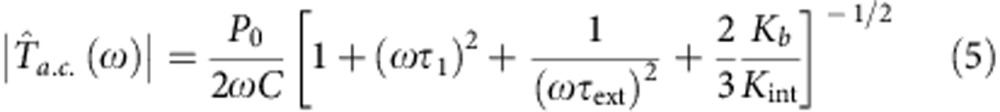



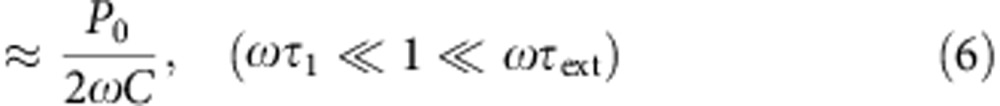


from which the heat capacity is obtained as 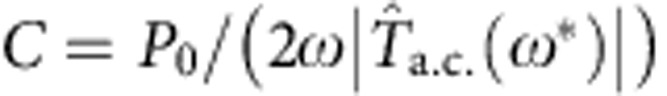, where ω* is within the sweet spot (*ωτ*_1_<<1<<*ωτ*_ext_). The inequalities in equation (6) determine the optimal frequency ω*.

**Figure 6:**
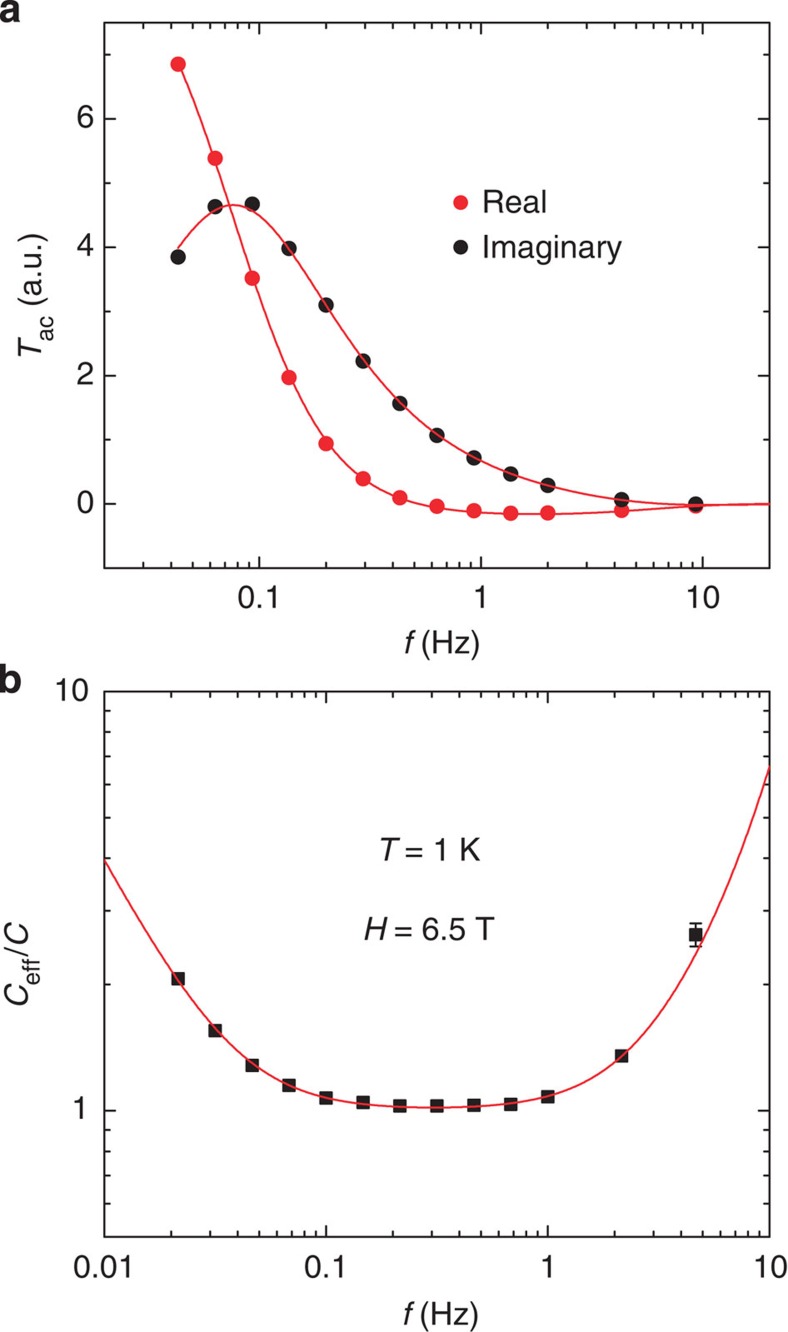
Spectra of the observed complex temperature and the inferred effective heat capacity at 1 K and 6.5 T. (**a**) The frequency dependence of the real (red dots) and imaginary part (black dots) of complex temperature 
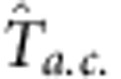
. The thin red curves show the fits to equation (3).(**b**) Frequency dependence of the effective heat capcacity (black squares) and the fit (red curve). The effective heat capacity C_eff_ coincides with the sample heat capacity 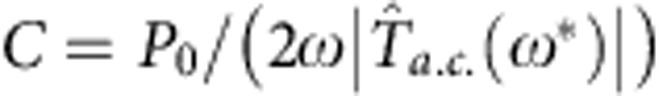 when the frequency ω* falls within the sweet spot *ωτ*_1_<<1<<*ωτ*_ext_. Throughout, *f*=*ω*/2*π* is the frequency of the a.c. power.

In the experiment, the measurements extended from 0.45 to 30 K in temperature and from 0 to 8 T in field. In each representative region of the *T*–*H* plane investigated, we have measured the frequency spectrum of the effective heat capacity 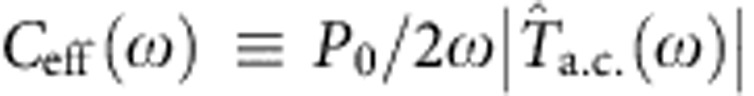. We carried out fits to the equations above, and found the optimal frequency ω* in each region of the *T*–*H* plane. The fit to one of the spectra is shown in Fig. 6b. The flat portion of the spectrum corresponds to the sweet spot in which *C* is identified with 
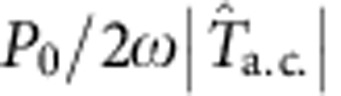
. The fits to the real and imaginary parts of equation (3) are shown in Fig. 6a.

**Figure 7:**
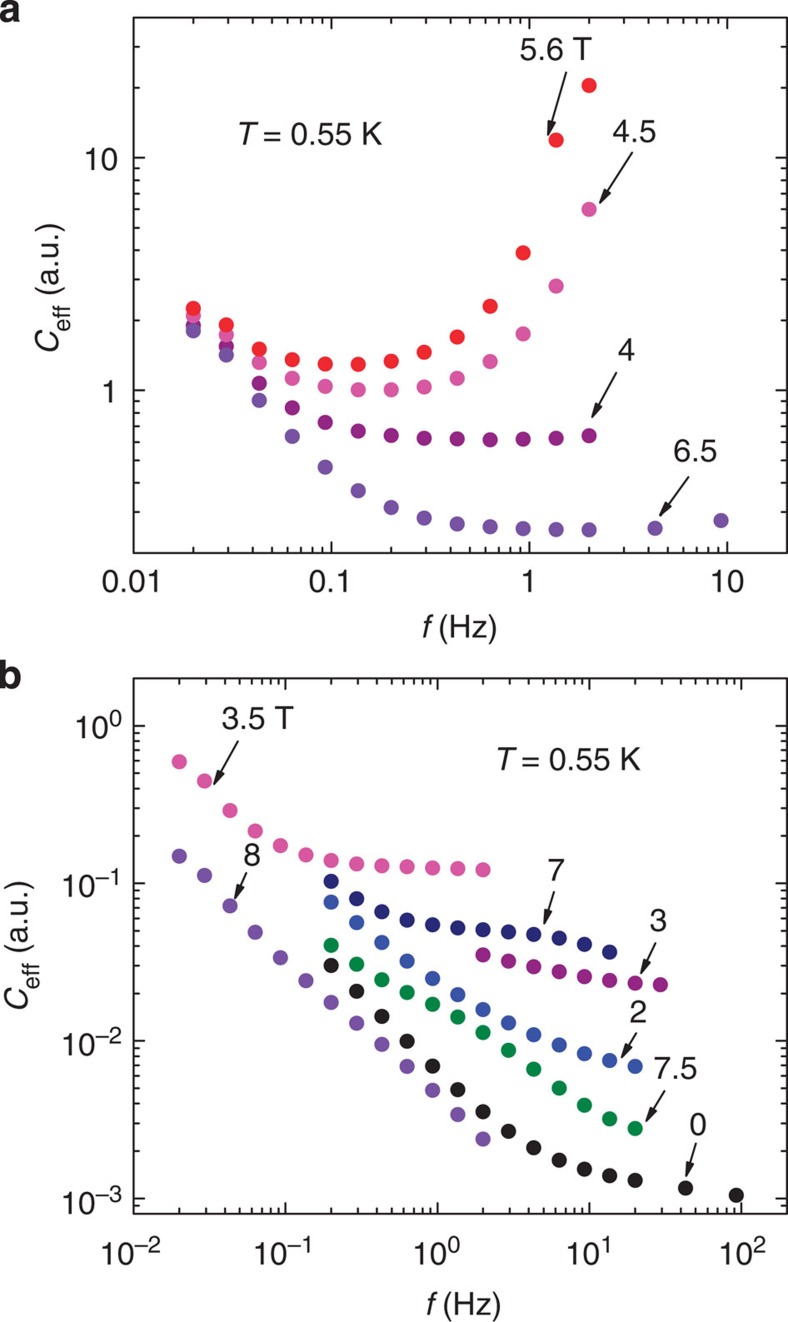
Spectra of the effective heat capacity *C*_eff_ at 0.55 K at selected fields. (**a**) Frequency dependence of the sweet spot near the quantum critical point. As the magnetic field reaches the quantum critical point, the sweet spot moves to lower frequencies, reflecting the increase of the heat capacity. (**b**) Glassy behaviour below 3.5 T and above 7 T. Effective heat capacity C_eff_ decreases monotonically as the frequency increases. No sweet spot was found in this field range. For clarity, the curves have been shifted vertically to avoid overlap.

Figure 7a displays the spectrum of *C*_eff_ at 0.55 K at several values of *H*. The evolution of the sweet spot as *H* varies is apparent. As *H* approaches the critical field *H*_c_, the sweet spot moves to lower frequencies, reflecting the increase of the heat capacity of the sample.

An important benefit of mapping the spectra over the entire *T*–*H* plane is that we can observe the onset of glassy behaviour. In the glass-like regions (which appear below 1 K in specific field ranges), the spectra decrease monotonically with increasing *ω*. The flat portion is not observed. Several traces for *H* below 3.5 T and above 7 T are shown in Fig. 7b (all curves are at 0.55 K). In these regimes (demarcated by the dashed curves in Fig. 2), the measured spectrum cannot be fitted to equation (3), so we cannot extract a value for *C*.

### Phonon contribution

**Figure 8:**
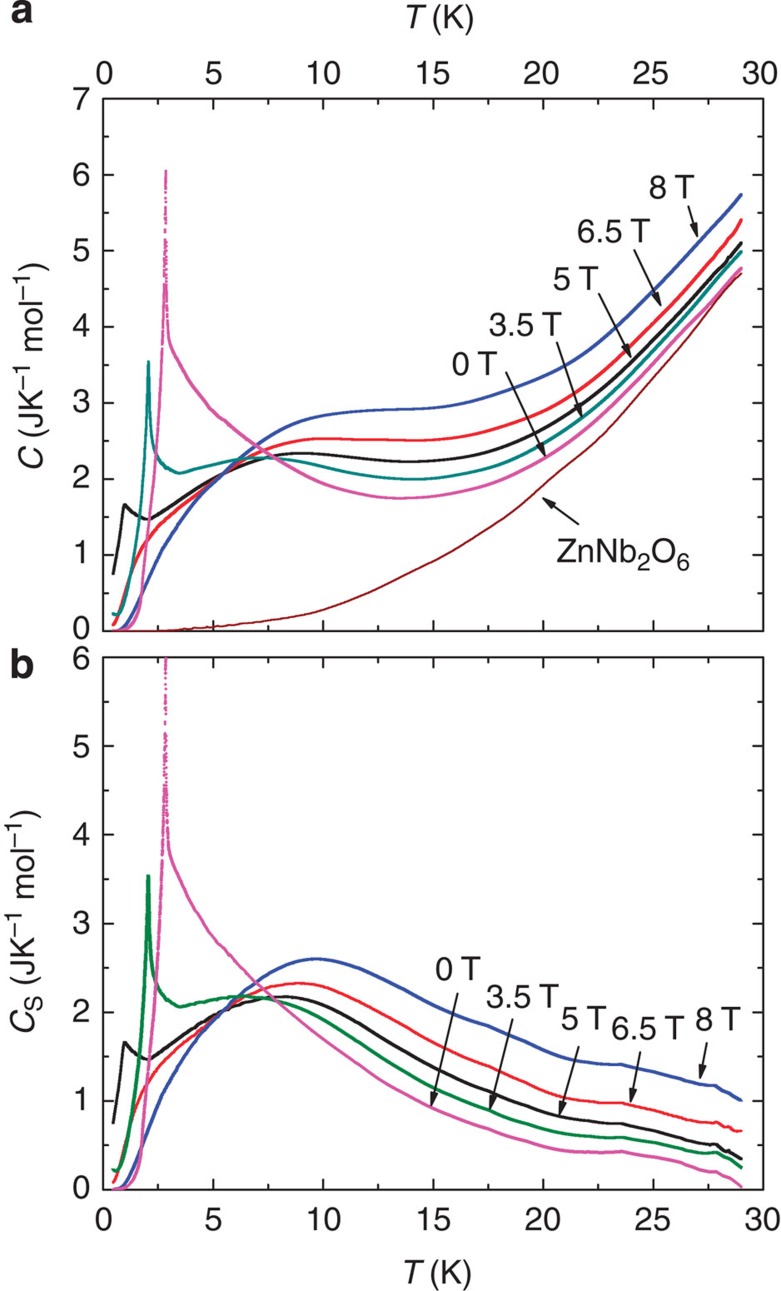
Behaviour of the heat capacity measured up to 30 K at selected magnetic fields. (**a**) The bold curves are the total heat capacity *C* measured below 30 K in fixed field *H*=0–8 T. The thin curve (wine coloured) shows the heat capacity of the nonmagnetic analogue ZnNb_2_O_6_ measured by Hanawa *et al*. We slightly rescaled their data by ∼13% to match our measured curves (the disagreement arises from uncertainties in estimating the crystal size). The phonon contribution to the total heat capacity is negligible below 4 K. (**b**) The spin heat capacity *C*_s_ obtained after subtraction of the phonon contribution at the selected *H*.

Below 4 K, the contribution of the phonon to the heat capacity is negligible. However, above 4 K, the phonon contribution to the observed *C* is substantial. To isolate the heat capacity of the spin degrees of freedom, we estimate the phonon contribution in CoNb_2_O_6_ as equivalent to the heat capacity in ZnNb_2_O_6_ measured by Hanawa *et al*.^12^. ZnNb_2_O_6_ is a nonmagnetic analogue of CoNb_2_O_6_ with nearly identical lattice structure. In Fig. 8a, the raw curves for CoNb_2_O_6_ (before the phonon subtraction) are plotted. The heat capacity of ZnNb_2_O_6_ is also plotted alongside with a slight rescaling (by 13%) to achieve asymptotic agreement with our zero-*H* curve when *T* exceeds 25 K. The rescaling is consistent with the combined experimental uncertainties in the two experiments. Curves of the spin contribution to the heat capacity *C*_s_(*T*), obtained after phonon subtraction, are plotted in Fig. 8b.

### Free-fermion solution

We use the free-fermion solution^15^,^17^ of the one-dimensional Transverse Ising Model to calculate the heat capacity of the spin d.o.f. The TIM Hamiltonian is


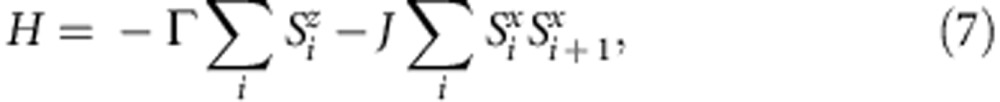


with Γ the applied transverse field (along 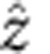) and *J* the easy-axis exchange (along 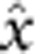). The spin operators *S*_*i*_^*x*^ and *S*_*i*_^*y*^, expressed in the combination


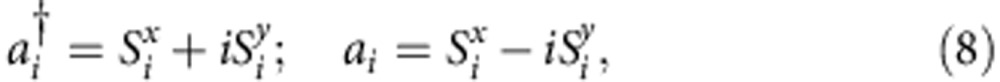


are converted by the Jordan–Wigner transformation into the fermion operators


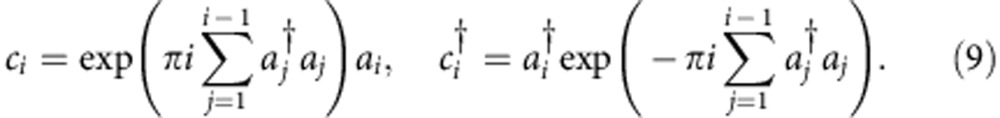


*H* is then reduced to terms bilinear in the fermion operators. A final Bogolyubov transformation to the new fermion operators 
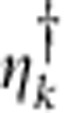
, *η*_*k*_ achieves diagonalization, and gives


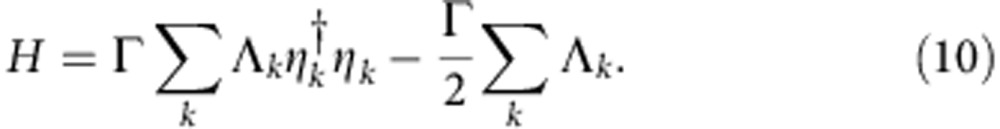


The free-fermion excitation energy is *ɛ*_*k*_=ΓΛ_*k*_, with


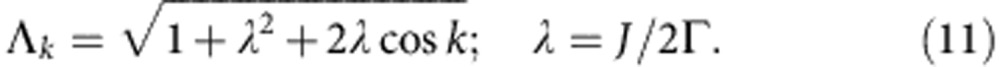


The free energy is given by


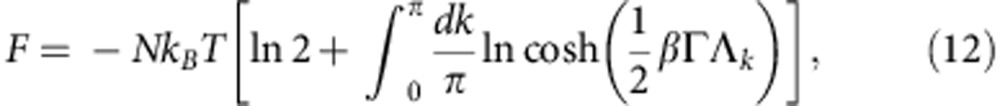


with *β*=1/(*k*_B_*T*). From *F*, we obtain the molar heat capacity in equation (2).

## Additional information

**How to cite this article:** Liang, T. *et al*. Heat capacity peak at the quantum critical point of the transverse Ising magnet CoNb_2_O_6_. *Nat. Commun.* 6:7611 doi: 10.1038/ncomms8611 (2015).
